# Inferring duplication episodes from unrooted gene trees

**DOI:** 10.1186/s12864-018-4623-z

**Published:** 2018-05-08

**Authors:** Jarosław Paszek, Paweł Górecki

**Affiliations:** Warsaw University, Faculty of Mathematics, Informatics and Mechanics, Banacha 2, Warsaw, 02-097 Poland

**Keywords:** Genomic duplication, Duplication episode, Minimum episodes problem, Reconciliation, Unrooted gene tree, Species tree

## Abstract

**Background:**

One of evolutionary molecular biology fundamental issues is to discover genomic duplication events and their correspondence to the species tree. Such events can be reconstructed by clustering single gene duplications inferred by reconciling a set of gene trees with a species tree.

**Results:**

Here we propose the first solutions to the genomic duplication problem in which every reconciliation with the minimal number of single gene duplications is allowed and the method of clustering called minimum episodes under the assumption that input gene trees are unrooted.

**Conclusions:**

We showed new theoretical properties of unrooted reconciliation for the duplication cost and apply them to design several exact and heuristic algorithms for solving the problem. Our evaluation study on empirical dataset confirmed several genomic duplication events from the literature and demonstrate that algorithms can be successfully applied.

## Background

The phenomenon of genomic duplication is fundamental to understand the evolution of life on Earth [1–5]. The research in phylogenetics focus on the way how the gene families and genomes evolve by discovering the locations of gene duplications. *Multiple gene duplications* occur when large parts of a genome are duplicated. In particular, the *whole-genome duplication* occurred for numerous species and had a crucial impact on the evolution of crops [6–9]. The studies of this phenomenon focus on detecting its occurrences as well as its influence on introgressing novel metabolic traits [10] or its association with periods of increased environmental stress [11]. The methods of detecting whole-genome duplications can be divided into three categories based on synteny and colinearity comparison of genomes [1, 12, 13], the estimation of the age distribution of paralogous gene pairs [3, 14], and phylogenetic tree inference [15–17].

The reconstruction of the evolution of individual genes has been thoroughly studied [18–22] also with the focus on gene trees [23–26], networks [27, 28], from the perspective of population genetics [29] or the evolution of entities (which can be genes, gene domains, or parts of genes) [30].

The reconciliation model, introduced by Goodman [31] and formalized by Page [18], interprets the differences between a gene tree and its species tree [32–34]. In this model, each node from a rooted gene family tree is mapped into the species tree and classified as a single gene duplication or related to a speciation event. In our work, we model a biologically consistent scenario as the embedding of a gene tree into a species tree which represents the location of evolutionary events in the species tree [35]. Identification of such a scenario is made by a function called duplication mapping that assigns a gene tree node, interpreted as a duplication event, to a node of a species tree [20, 36–43]. Reconciliation becomes complex when considering multiple gene duplications. The general formulation is as follows: *given a set of gene trees and a species treefind evolutionary scenarios for the collection of gene trees that yields the minimal number of multiple gene duplication events* [44]. Two fundamental issues arise when dealing with multiple gene duplications: a model of allowed evolutionary scenarios [20, 44, 45] and the rules of clustering gene duplications from gene trees into multiple duplication events. We distinguish three variants of problems depending on the clustering: *episode clustering (EC)* [20, 37, 38], gene duplication clustering (GD) [45], and *minimum episodes (ME)*. EC is to find scenarios having the minimal number of locations of duplication episodes in a species tree. EC for rooted gene trees has a linear time solution [42], while for unrooted trees an FPT algorithm is known [36]. GD is similar to EC with the difference that a cluster cannot have two gene duplications from the same tree. In ME a duplication and its ancestor duplication cannot be clustered together [20, 38]. The first polynomial time algorithm for ME with rooted gene trees, called *RME*, under the model from [20] was proposed in [38], whereas the optimal linear time algorithm in [41, 42]. The concept of assigning every duplication to an interval of allowed locations in a species tree was introduced in [46] in a more general framework without the requirement that the intervals induce a biologically consistent scenario. The naïve implementation of the iterative algorithm from [46] has cubic time complexity. The solution to RME for a variety of models was presented in [44]. In particular, the algorithm proposed in [44] solves RME in linear time.

*Our contribution.* We propose the solution to the *unrooted minimum episodes problem*, *UME*, in which allowed scenarios have the minimal number of gene duplications [36]. According to our knowledge, the complexity of UME is unknown. We expanded the theory of unrooted reconciliation by presenting new properties of the *plateau* which is the subtree of an unrooted gene tree containing edges whose rootings have the minimal duplication cost. Next, we show that these properties lead to a decomposition of an unrooted gene tree that allows limiting the possible search space significantly. We show that every instance of UME can be transformed into at most 5^*k*^ “simpler” instances that can be solved in linear time, where *k* is bounded above by special cases of S2 stars [47] in input trees. Next, we propose two linear time algorithms for computing bounds of the score. Finally, for the case when *k* is large, we propose an efficient heuristic algorithm, which in practice allows solving exactly empirical instances consisting of thousands of unrooted gene trees. Also, we present an evaluation of several empirical datasets.

## Methods

### Basic notation

Let *S* denote a *species tree* which is a rooted binary tree with leaves uniquely labeled by the names of species. We assume that *S* is fixed throughout this work. A *rooted gene tree* is a rooted binary tree with leaves labeled by the names of species. The rooted tree (*T*_1_,*T*_2_) has two subtrees *T*_1_ and *T*_2_ whose roots are the children of the tree root. Additionally, for nodes *a* and *b*, we write $a \preceq b$ when *a* and *b* are on the same path from the root, with *b* being closer to the root than *a*. Notation $a \prec b$ means that $a \preceq b$ and *a*≠*b*. The root of a tree *T* we denote by *root*(*T*). By *T*_*v*_, we denote the subtree of *T* rooted at *v*. A cluster for a node *v* is the set of all species present in *T*_*v*_.

Let *T*=〈*V*_*T*_,*E*_*T*_〉 be a rooted gene tree such that the set of species present in *T* is a subset of the set of species present in *S*. *The least common ancestor (lca) mapping*, M_*T*_:*V*_*T*_→*V*_*S*_, is defined as follows. If *v* is a leaf in *T* then M_*T*_(*v*) is the leaf in *S* labeled by the label of *v*. For an internal node *v* in *T* having two children *a* and *b*, mapping M_*T*_(*v*) is the least common ancestor of M_*T*_(*a*) and M_*T*_(*b*) in *S*. An internal node *g*∈*V*_*T*_ is called a *duplication* if M_*T*_(*g*)=M_*T*_(*a*) for a child *a* of *g*. *The duplication cost*, the total number of duplications in *T*, is denoted by D(*T*,*S*). Every non-duplication node of *T* we call a *speciation* (including leaves).

### Evolutionary scenarios

Here, we present the model of DLS trees [35] that will be used to represent evolutionary scenarios. A *DLS tree* is a binary tree having two types of internal nodes, that denote *speciation* and *duplication* events, and two types of leaves that denote *gene loss* and *gene sequences*. DLS trees are defined as follows [44]:

1. *a* is a single-noded DLS tree denoting a *gene sequence* from the species *a*,
2. *A*- is a single-noded DLS tree denoting a *lost gene* lineage, where *A* is a non-empty set of species,
3. (*R*_1_,*R*_2_)+ is a DLS tree whose root is a duplication node and its children are DLS trees *R*_1_ and *R*_2_ such that the set of species present in *R*_1_ and the set of species present in *R*_2_ are equal,
4. (*R*_1_,*R*_2_)∼ is a DLS tree whose root represents a speciation and its children are DLS trees *R*_1_ and *R*_2_ such that the set of species present in *R*_1_ and the set of species present in *R*_2_ are disjoint.

Let *T* be a DLS-tree with at least one gene sequence. A gene tree can be extracted from *T* by contracting nodes of degree 2 from the smallest subgraph of *T* containing all gene sequences. Such an operation will be denoted by *gt*(*T*).

**Fig. 1:**
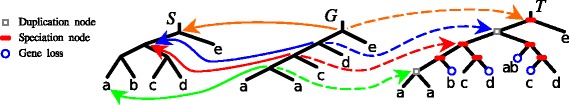
An example of scenario *T* for a gene tree *G* and a species tree *S* and two corresponding mappings: *ξ*:*G*→*T* and $F_{T} \colon G \rightarrow S$ shown for internal nodes of *G*. Here, *T*=(((((a,a)+,b-) ∼,(c,d-) ∼) ∼,(ab-,(c-,d) ∼) ∼)+,e) ∼, note that *gt*(*T*)=*G*

We say that a DLS-tree *T* is a *scenario* for a gene tree *G* and a species tree *S* if *gt*(*T*)=*G*, and *T* is *compatible* with *S*, that is, every cluster of *T* is present in *S*. In such a case, every node *g* in *G* uniquely corresponds to a node in *T* denoted by *ξ*(*g*). We can define mappings $\xi \colon G \rightarrow T$ and $F_{T} \colon G \rightarrow S$, such that *F*_*T*_(*g*) is the node in *S* whose cluster equals the cluster of *ξ*(*g*). An example is depicted in Fig. 1.

### Unrooted reconciliation

*The unrooted gene tree* is an undirected acyclic connected graph in which each internal node has degree 3, and the leaves are labeled by the names of species. The rooting of an unrooted gene tree *U*=〈*V*_*U*_,*E*_*U*_〉 obtained from *U* by placing the root on an edge *e*∈*E*_*U*_ is denoted by *U*_*e*_. Such a rooting induces the duplication cost D(*U*_*e*_,*S*). An edge *e* is called *optimal* if D(*U*_*e*_,*S*) is minimal in the set of all rootings of *U*. It is known that the set of optimal edges, called the *plateau*, is a full subtree of *U* [47, 48]. In this article, the notion of the plateau is used exclusively with the duplication cost. In literature, it is often called D-plateau in order to distinguish between plateaus for other costs, e.g. DL-plateau [48]. In this work, the subtree induced by the set of all optimal edges will be denoted by *U*^∗^. For *X*, the set of edges of unrooted tree *U*, by *U*|_*X*_ we denote the smallest subgraph of *U* containing all edges from *X*.

**Fig. 2:**
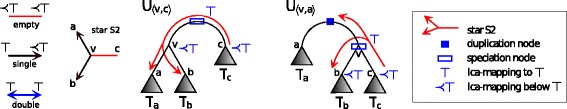
Types of edges, star S2, and two rootings of an unrooted gene tree *U*: on the empty edge 〈*v*,*c*〉 and on the single edge 〈*v*,*a*〉. Here, ⊤ denotes the root of *S*

Without loss of generality, we assume that every root of a gene tree is mapped into the root of *S*, and both trees are non-trivial. An edge *e*=〈*v*,*w*〉 of *U* can be classified as one of three following types: (a) *empty* if the root of *U*_*e*_ is a speciation, i.e., M_*e*_(*v*)≠*root*(*S*)≠M_*e*_(*w*), (b) *double* if M_*e*_(*v*)=*root*(*S*)=M_*e*_(*w*), and (c) *single* otherwise, where *M*_*e*_ is the lca-mapping between *U*_*e*_ and *S*. Let *v* be an internal node of *U*, then a *star* with the *centerv* consists of three edges, sharing *v*. There are five possible types of stars present in unrooted gene trees [47, 48], however, in this article we only use the star called *S*2 having one empty edge. In such a case the remaining edges are single, and by using the notation from Fig. 2, for *x*∈{*a*,*b*} we have that $\mathsf {M}_{U_{\langle {v,x}\rangle }}(x) \neq \mathsf {root}(S) = \mathsf {M}_{U_{\langle {v,x}\rangle }}(v)$.

It follows from unrooted reconciliation that plateau has either exactly one empty edge or at least one double edge [47]. We say that a node is a *super-duplication* (respectively, a *super-speciation*) if it is a duplication (respectively, a speciation) in every rooting with the minimal duplication cost.

#### **Lemma 1**

[adapted from [36]] Assume that an unrooted tree has a double edge. Then, every leaf of the plateau is a super-speciation, and every internal node of the plateau is a super-duplication.

On the other hand, when there is an empty edge in an unrooted tree, we have:

#### **Lemma 2**

Let *U* be an unrooted gene tree with an empty edge *e*. A node incident to *e* is a speciation in *U*_*e*_ if and only if it is a leaf of the plateau.

#### *Proof*

We use the notation from Fig. 2 where *e* is 〈*v*,*c*〉. We may assume that *c* is an internal node of *U*; otherwise, we have a trivial case where *c* is a leaf in the rooting of *U* which is a speciation. Thus, we have two S2 stars sharing the empty edge. $(\Leftarrow)$ Without loss of generality, we may assume that *v* is a leaf of *U*^∗^. If *v* is not a speciation in *U*_〈*v*,*c*〉_ then it is a duplication. From the definition of the empty edge, the root of *U*_〈*v*,*c*〉_ and *v* in *U*_〈*v*,*a*〉_ are speciation nodes. Moreover, the node *v* in *U*_〈*v*,*a*〉_ is mapped to *root*(*S*) thus the root of *U*_〈*v*,*a*〉_ is a duplication. Both rootings *U*_〈*v*,*c*〉_ and *U*_〈*v*,*a*〉_, have the same number of duplications having the same setting of duplications in subtrees *T*_*a*_,*T*_*b*_ and *T*_*c*_ as indicated in Fig. 2. Hence, 〈*v*,*a*〉 is a *U*^∗^ edge, a contradiction. $(\Rightarrow)$ The proof is similar to the first case. □

The conclusion from the above Lemma 2 is that either only empty edge or the whole S2 star is included in the plateau. Moreover, we can describe the plateau having an empty edge by the following lemma:

#### **Lemma 3**

If the unrooted gene tree has an empty edge then every leaf of the plateau is a super-speciation, and every internal node of the plateau not incident to an empty edge is a super-duplication.

#### *Proof*

For the first part of the proof, let assume that *v* is a leaf of *U*^∗^ which consists of 〈*v*,*c*〉 edge. Assume that *v* is a duplication in some plateau rooting. Then, the subtree *T*_*v*_ in this rooting is also a subtree in all plateau rootings because *v* is a leaf of *U*^∗^. Hence, *v* is a super-duplication. If 〈*v*,*c*〉 is an empty edge we have a contradiction from Lemma 2. Assume that 〈*v*,*c*〉 is non-empty. The edge 〈*v*,*a*〉 does not belong to *U*^∗^. Therefore, the rooting *U*_〈*v*,*a*〉_ has more duplications than *U*_〈*v*,*c*〉_. Hence, *U*_〈*v*,*a*〉_ has two duplications in *v* and in the root. Therefore, the root of *U*_〈*v*,*c*〉_ is not a duplication. However, this is possible only when *T*_*a*_ and *T*_*v*_ are mapped below the *root*(*S*), thus the 〈*v*,*c*〉 is an empty edge, a contradiction. For the next part of the proof, if *U*^∗^ consists of exactly one empty edge then the property holds trivially. Let assume that the *U*^∗^ has more than one edge. We show that every internal node *v* of *U*^∗^, that is, not incident to an empty edge is a super-duplication. Let us consider a path $p = v_{1}, v_{2}, \dots, v_{n}$ (*n*>1) consisting of nodes not incident with the empty edge connecting *v*=*v*_1_ with a leaf *v*_*n*_ of *U*^∗^. Hence, when rooting on *p*, *v* is mapped to *root*(*S*) as it is the ancestor of nodes incident with the empty edge. Moreover, when rooting on 〈*v*_*n*−1_,*v*_*n*_〉, we have *n* gene duplications: for $v_{1}, v_{2}, \dots, v_{n-1}$ and one for the root. All edges from *p* are elements of *U*^∗^, thus moving the root to other edges on *p* will preserve the total number of gene duplications. We showed that the first *n*−1 nodes on *p* are duplications for every rooting placed on this path. If *v* is incident to an empty edge, it is a speciation mapped to the *root*(*S*) when rooting on *p*. When rooting on an empty edge, the root is a speciation. Moreover, from Lemma 2 a child of the root is a duplication if it is an internal node of *U*^∗^. Hence, all plateau rootings have the same number of duplications equalling the number of internal nodes of *U*^∗^. When rooting on an empty edge, the root is a speciation and all internal nodes of *U*^∗^ are duplications. Otherwise, if we place the root on the edge from *U*^∗^, the root is a duplication node and the only speciation is that node among nodes incident to an empty edge which is an ancestor to the other. □

### Clustering Duplications: Minimum Episodes Problems

We define the cost determining the number of multiple gene duplication episodes for a set of evolutionary scenarios. Let $\mathcal {R}$ be a set of scenarios compatible with *S*. We say that duplications *d* and $d^{\prime }$ from $\mathcal R$ are *clusterable*, denoted $d \ \sim _{c}\ d^{\prime }$, iff (1) *d* and $d^{\prime }$ have the same cluster and (2) if *d* and $d^{\prime }$ are present in the same DLS-tree then either *d* and $d^{\prime }$ are incomparable or equal. Then, the minimum number of duplication episodes for $\mathcal {R}$, denoted $\mathsf {MES}({\mathcal R},S)$, is the size of the smallest partition of the set of all duplication nodes from $\mathcal R$ induced by an equivalence relation contained in ∼_*c*_.

It can be shown that for a collection $\mathcal {R}$ of scenarios compatible with a species tree *S*,

1 $$  \mathsf{MES}({\mathcal R},S)=\sum_{v \in V_{S}} \max_{T \in {\mathcal R}} \mathsf{duppath}(T,v),  $$

where *duppath*(*T*,*v*) is the maximal (node) length of the path in *T* that consists of all comparable duplication nodes whose cluster equals the cluster of *v* [44].

Let $\mathcal {A}(G, S)$ be the set of all scenarios for a rooted gene tree *G* and a species tree *S* having the minimal number of gene duplications. Every element of $\mathcal {A}(G, S)$ will be referred to as an *allowed scenario*. Here, allowed scenarios are defined as in [36], for the comprehensive overview see [44]. Now, we formulate the general problem in which the input consists of mixed types of gene trees: rooted and unrooted.

#### **Problem 1**

[General Minimum Episodes, GME] *Given* a collection of gene trees (rooted or not) $\mathcal {U}=\left \{U^{1},U^{2},\dots,U^{n}\right \}$ and a species tree *S*. *Computeminimum episodes score*$\mathsf {ME}(\mathcal {U},S)$, or *ME* score, as the minimal value of $\mathsf {MES}(\{ R_{i} \}_{i=1,2,\dots,n},S)$ in the sets of scenarios *R*_*i*_ such that $R_{i} \in \mathcal {A}(U^{i},S)$ if *U*^*i*^ is rooted or $R_{i} \in \mathcal {A}\left (U^{i}_{e},S\right)$ if *U*^*i*^ is unrooted, where *e* is an optimal edge.

Observe that we allow only scenarios that preserve the minimal number of gene duplications. We distinguish two variants of *GME* Problem: unrooted minimum episodes (*UME*) and rooted minimum episodes (*RME*) in which the instances consist entirely of unrooted and rooted gene trees, respectively. *RME* Problem has a linear time and space solution [44]. See also [38, 42] for more details on *RME* Problem.

### Unrooted tree decomposition

In this section, we show that every unrooted gene tree can be decomposed into a set of trees having at most one unrooted tree with a simplified structure allowing to solve *UME* more efficiently. We start with the following observation.

#### **Lemma 4**

Let *U* be an unrooted gene tree and *T* be a rooted subtree of *U* rooted at *v*. Let $X \subseteq U^{*}$ such that

- *X* is disjoint with *V*_*T*_∖{*v*},
- *v* is a speciation in every scenario from $\mathcal {A}(U_{e},S)$ for all *e*∈*E*_*X*_.

Then, for any set of scenarios $\mathcal {X}$:

2 $$ \begin{aligned} \min_{R \in \mathcal{A}(U_{e},S), e \in E_{X}} \mathsf{MES}({\mathcal X} \cup \{R\},S)= \\ \min_{\substack{R^{\prime} \in \mathcal{A}(U\prime_{e},S), e \in E_{X}, \\ R^{\prime\prime} \in \mathcal{A}(T,S)}} \mathsf{MES}({\mathcal X} \cup \{R^{\prime},R^{\prime\prime}\},S),  \end{aligned}  $$

where $U^{\prime }_{e}$ is the unrooted tree obtained from *U* by replacing *T* with *S*(*M*(*v*)).

#### *Proof*

In every allowed scenario *R* from the left side, $F_{U_{e}}(v)$ is a speciation node. Thus, scenarios $R^{\prime }$ and $R^{\prime \prime }$ can be obtained from *R* as follows: $R^{\prime \prime }$ is the subtree rooted at $F_{U_{e}}(v)$ in *R*, while $R^{\prime }$ is obtained from *R* by replacing the subtree with the copy of *S*(*M*(*v*)), where every internal node is a speciation. Such a transformation is a bijection that preserves the clusterability of duplication nodes. We omit technical details. □

**Fig. 3:**
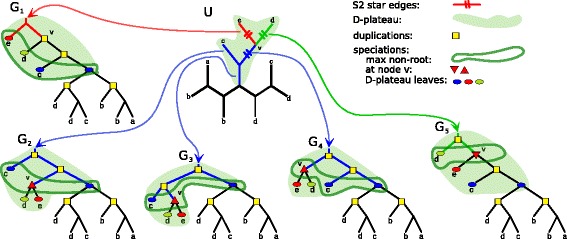
Equivalence relation ∼. An example of an unrooted gene tree *U* with one S2 star and all plateau rootings reconciled with a species tree S = (((a,b),(c,d)),e). *U*^∗^ contains five edges and induces three ∼-equivalence classes. The first consists of an empty edge 〈*e*,*v*〉, the second of 〈*d*,*v*〉 while the last class consists of the remaining three edges. These three classes induce rootings {*G*_1_}, {*G*_5_} and {*G*_2_,*G*_3_,*G*_4_}, respectively. Observe, that $\widetilde {G}_{2} = \widetilde {G}_{3} = \widetilde {G}_{4}$ consist of a subset of *U*^∗^ leaves and a speciation (different for each class) at node *v* which is a center of S2 star

Given a species tree *S* and a rooted tree *G* by $\widetilde {G}$ we denote the set of all $\preceq $-maximal elements in the set of all non-root speciation nodes from *G*. Lets ∼ be a relation on edges of *U*^∗^ for an unrooted gene tree *U* such that $e \sim e^{\prime }$ if $\widetilde {U}_{e} = \widetilde {U}_{e^{\prime }}$. It should be clear that ∼ is an equivalence relation. The set of equivalence classes of this relation we denote by *U*^∗^ /_∼_. An example is depicted in Fig. 3.

#### **Lemma 5**

If an empty edge is present in an unrooted gene tree then every plateau edge present in S2 star uniquely defines one ∼-equivalence class. Otherwise, the tree has exactly one ∼-equivalence class.

#### *Proof*

Let *U* be an unrooted gene tree. We have two cases: (a) either *U* has a double edge or (b) *U* has an empty edge. In the case (a), it follows from Lemma 1, that $\widetilde {U}_{e}$ consists of all *U*^∗^ leaves for every *e* from *U*^∗^. Thus, we have one equivalence class consisting of all *U*^∗^ edges. Let use the notation from Fig. 2. For the case (b), from the proof of Lemma 3 we conclude that for the empty edge 〈*v*,*c*〉 the set $\widetilde {U}_{\langle {v,c}\rangle }$ consists of all *U*^∗^ leaves. Moreover, from the conclusion from the proof of Lemma 2, there are 0,2 or 4 single edges in *U*^∗^ present in S2 stars. Let 〈*v*,*a*〉 be such an edge. The set $\widetilde {U}_{\langle {v,a}\rangle }$ consists of: (a) *v* which is the root of the subtree *T*_*v*_=(*T*_*b*_,*T*_*c*_) and thus it is a speciation (it maps to *root*(*S*) and both its children map below the *root*(*S*)) and (b) all leaves of *U*^∗^ present in *T*_*a*_. From Lemma 3 for every edge *e* of *U*^∗^ present in *T*_*a*_, we have $\widetilde {U}_{e} = \widetilde {U}_{\langle {v,a}\rangle }$. Summing up there can be 1,3 or 5 ∼-equivalence classes uniquely defined by every edge of *U*^∗^ present in S2 star (see Fig. 3). □

If an empty edge is an element of a class *X*∈*U*∗/_∼_, *X* will be called *plain*. Otherwise, we call *Xcomplex*.

#### **Lemma 6**

If *X*∈*U*∗/_∼_ is complex then the leaves from *U*|_*X*_ are speciations in every tree *U*_*e*_ for every *e* in *X*.

#### *Proof*

*U* has either an empty or a double edge. The leaves of *U*^∗^ are super-speciations from Lemmas 1 and 3. If *U* has a double edge, then there is only one ∼-equivalence class (Lemma 5) and every leaf *v* of *U*|_*X*_ is also a leaf in *U*^∗^. If *U* has an empty edge, say *e*, then there are 0, 2 or 4 classes *X* disjoint with {*e*}. For all of them the set of the leaves of *U*|_*X*_ consists of a subset of the leaves of *U*^∗^ (disjoint with subsets corresponding to other classes see Fig. 3) and a node *v* which is the center of a star S2 and a speciation when rooting on edges from *X* (see the proof of Lemma 5). □

#### **Definition 1**

[Unrooted Decomposition] Let *U* be an unrooted gene tree, and *X*∈*U*∗/_∼_, then:

- If *X* has an empty edge *e* then *Δ*(*U*,*X*)={*U*_*e*_}.
- Otherwise, *Δ*(*U*,*X*) is the set of all maximal subtrees *T*_*v*_ of *U* such that *v* is a leaf of *U*|_*X*_ and $T_{v} \cap U|_{X} = \{v\}$.

For a complex class *X*, *U*^*X*^ denotes a tree obtained from *U*|_*X*_ by replacing every leaf *v* with the subtree *S*(M(*root*(*T*_*v*_))). For example, for the largest class *X* from Fig. 3, we have: $\Delta (U,X) = \{ c,\ (d,e),\ \left ((a,b),b),((c,d),d)\right) \}$ and *U*^*X*^=((((*a*,*b*),(*c*,*d*)),*e*),((*a*,*b*),(*c*,*d*)),*c*).

The intuition is that *Δ*(*U*,*X*) is the set of rooted trees *T* induced by *X* with the following properties: (a) the root of *T* is a speciation, and (b) *T* is a subtree present in all rootings induced by *X*. For example, when we consider an empty class there is only one possible rooting *U*_*e*_. Lemma 6 describes the properties of *Δ*(*U*,*X*) for a complex class *X*. Finally, for an unrooted tree *U* we have the following formula:

#### **Lemma 7**

[Decomposition Lemma] For a given set of input gene trees $\mathcal {G}$, an input unrooted gene tree *U* and a species tree *S* we have, $\mathsf {ME}(\mathcal {G} \cup \{ U\},S) = \min _{X \in U{*}/_{\sim }} \left \{\begin {array}{cc} \mathsf {ME}(\mathcal {G} \cup \{U_{e}\}) & \\ & {} \text {if}\ X=\{e\}\ \text {and}\ e\ \text {is empty,}\\ \min _{e \in X} \mathsf {ME}(\mathcal {G} & \hspace {-10pt} \cup \left \{ U^{X}_{e} \right \} \cup \Delta (U,X), S) \\ & {\kern -2.4cm} otherwise. \end {array}\right.$

#### *Proof*

Let us consider the set of allowed DLS scenarios induced by rootings of edges from each *X*∈*U*∗/_∼_. If *X* is plain, then the set is $\mathcal {A}(U_{e},S)$. If *X* is complex, then by Lemma 6, *X* and every leaf *v* from *U*|_*X*_, satisfies assumptions from Lemma 4. Thus, the subtree of *U* disjoint with *X*∖{*v*} can be detached and replaced by *S*(*M*(*v*)) in *U*. By Lemma 4 the *MES* score is preserved. The rest follows by induction on the set of leaves *v*, where we show that the unrooted tree after all transformations is *U*^*X*^ and the set of detached subtrees is *Δ*(*U*,*X*). □

## Algorithms

### Solution to RME

We start with the linear time algorithm for *RME* from [44] adapted to the model of allowed scenarios presented here.



For the input consisting of rooted gene trees, every duplication *d* is associated with the interval consisting of all possible locations of *d* in the species tree. Our model of allowed scenarios is equivalent to the model from [44], in which I(*d*) is an interval defined by a pair 〈M(*d*),*s*〉, where *s*≽M(*d*) is the child of M(*g*) such that *g* is the lowest speciation satisfying *g*≻*d*, or *s* is the root if such a speciation does not exist. Algorithm 1 is a greedy bottom-up algorithm that iteratively assigns duplications to the top-end of intervals. In every step, it finds the lowest top node *s* of available intervals and assigns to *s* all duplications *d* having $\max \mathsf {I}(d)$ equal to *s*. Additionally, the algorithm assigns other duplications to *s* but only if the *ME* score is not increased, which is controlled by *λ*(*s*). For details please refer to [44].

### Exact solution to UME

A naïve solution to UME is to run RME algorithm for every combination of plateau rootings from input gene trees. In many cases the plateau can be large, hence, the time complexity of such a solution is $O(\prod _{i}|U_{i}|(\sum _{i}|U_{i}|+|S|))$. Here, we propose an algorithm based on Lemma 7 to limit the cases that have to be checked to the number of classes of ∼ relation.



#### **Lemma 8**

[Correctness of *gnaw*] Let *U* be an unrooted gene tree and *X* be a complex class. Let $\mathcal {X}_{r}$ be a set of rooted gene trees *T* such that the root of every *T* is a speciation. Let *me*(*u*,*v*)=〈*s*,*n*〉, in a call of *gnaw* with *U*^*X*^ and $\mathcal {X}_{r}$, such that *v* is internal in *X*. Then,

- for every rooting $U^{X}_{e}$ such that *e*∈*X*, and having *v* below the root, if Algorithm *1* (RME) is executed for $\mathcal {X}_{r} \cup \ \left \{U^{X}_{e}\right \}$, then *v* is assigned to a node *s* and *n*=*level*_*s*_(*d*),
- the call of *gnaw* returns $\min _{e \in X} ME\left (\mathcal {X}_{r} \cup \left \{U^{X}_{e}\right \}\right)$.

#### *Proof*

**Fig. 4:**
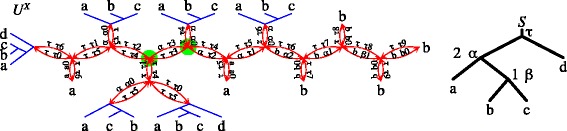
Illustration of *gnaw* for *U* with a double edge. Here, *τ* denotes the root of *S*. Assume that *S* has two positive lambda’s computed in line 7 by Algorithm 1: *λ*(*α*)=2 and *λ*(*β*)=1. Every edge *e*=〈*u*,*v*〉 of the plateau is split into two directed edges: 〈*u*,*v*〉 and 〈*v*,*u*〉. Each directed edge 〈*u*,*v*〉 is decorated with the lca-mapping *M*_〈*u*,*v*〉_(*v*) and *me*(*u*,*v*). For example, *τ*6 denotes the lca-mapping to *τ* and *me*(*u*,*v*)=〈*τ*,6〉. Here, *gnaw* returns 3+1+3 induced by the marked edge

First, observe that every call of *gnaw* satisfies the assumptions (see Def. 1). Assume that *e*∈*X*. Then, by the properties of a complex class *X*, we have in $U^{X}_{e}$ that the root and all internal nodes of *X*, are duplications, while all leaves of *X* are speciations. Let $X^{\prime }_{e}$ be the set of duplication nodes from *X* including the root. Thus, for every $d \in X^{\prime }_{e}$, we have I(*d*)=〈*M*_*e*_(*v*),*root*(*S*)〉, where *M*_*e*_ is the lca-mapping from $U^{X}_{e}$ to *S*. Hence, all duplications from $\mathcal {X}_{r}$ have the top interval node below the root, therefore, if Algorithm 1 (RME) would be called with the input consisting of $\mathcal {X}_{r} \cup \ \left \{U^{X}_{e}\right \}$, then, for *v* being the root of *S* (in line 2 of Algorithm 1), all $\mathcal {X}_{r}$ duplications are already processed. Additionally, a duplication *d* from $X^{\prime }_{e}$ can be assigned earlier to a node *v*≽*M*_*e*_(*d*) only in step 5, if the condition is satisfied. Thus, we can separate the process of RME computation for $\mathcal {X}_{r}$ (line 7 of Algorithm 2) and the rootings of *U*^*X*^. Furthermore, processing *U*^*X*^ can be done collectively for all rootings from *X*, by using a dynamic programming that jointly executes the assignment operation. Note, that in line 11 the first elements of *me*(*x*,*u*) and *me*(*y*,*u*) are comparable (i.e., *u* is a duplication), therefore, $\max $ is well defined by using lexicographical order. The proof of the first part follows by induction, in which a node in a rooted subtree of *U*^*X*^ is assigned to the first next free “slot” in a species node. Such a slot can be located by using *next*. When all slots of non-root nodes are occupied then duplications have to be assigned to the root. Such assignments create new episode events. Thus, the score of every rooting placed on *e*={*u*,*v*} can be easily computed by verifying if such additional episodes were created. This information is stored for the two subtrees of the root in *me*(*u*,*v*) and *me*(*v*,*u*), respectively, i.e., if *me*(*u*,*v*)=〈*root*(*S*),*n*〉, then *n* additional episodes are required. This value for both subtrees is stored in *m*_*e*_. Note that, $\max $ in line 12 is well defined, otherwise, *X* cannot be complex. Additionally, the root of $U^{X}_{e}$ creates one more episode. Therefore, the score returned by *gnaw* consists of *r* (from rooted trees), the minimal value of *m*_*e*_ (the contribution of *X*) and 1 (the root duplication). An example is depicted in Fig. 4. □

#### **Lemma 9**

[Correctness] Given a collection of unrooted gene trees $\mathcal U$ and a species tree *S*, Algorithm *2* returns $\mathsf {ME}(\mathcal {U},S)$.

#### *Proof*

The proof follows from Decomposition Lemma 4 and Lemma 8. □

#### **Lemma 10**

[Complexity of Exact UME] Algorithm *2* requires $O\left (\left (|S|+\sum _{i}|U_{i}|\right)5^{k}\right)$ time and $O\left (\sum _{i}|U_{i}|+|S|\right)$ space, where *k* is the number of gene trees with S2 star having more than one class of *U*∗/_∼_.

#### *Proof*

*Time:* The number of iterations of the main loop is bounded above by 5^*k*^. Locating classes of ∼ and transforming trees can be done in linear time. Each call of function *gnaw* requires $O\left (\sum _{T \in \mathcal {X}_{r}}|T|+|U^{X}|\right)$ time. *Space:* It follows from the complexity of Algorithm 1 and *gnaw*. □

### Solving hard instances

In this section, we propose several alternative solutions to our problem designed to cope with hard instances of ME Problem. For example, when the input consists of thousands of trees, it is more likely that *k* is large enough (e.g., for *k*≥20) to prohibit applications of Algorithm 2.

The first approach, presented in Algorithms 3 and 4, is to decrease the search space by introducing the lower and upper bounds on the optimal solution in a similar way that we proposed in [44]. In these algorithms we define function *gnawrooting*, being a variant of *gnaw* from Algorithm 2., that instead of the minimal score it returns the corresponding optimal rooting of the input gene tree.



#### **Lemma 11**

Algorithm *3* computes the lower bound of ME score in $O\left (|S|+\sum _{i}|U_{i}|\right)$ time and space.

#### *Proof*

Algorithm 3 computes the score from a set of input gene trees as follows. For each gene tree *U*:

- If *U*∗/_∼_ contains exactly one class then decompose the tree similarly to Algorithm 2, i.e., incorporate all duplications from *U* into the clustering space.
- Otherwise, ignore every duplication located in the plateau. In other words, to preserve all non-plateau duplications, it is sufficient to extract all (rooted) subtrees of *U* obtained from *U* by removing all internal nodes of the plateau.

Having this, we conclude that the size of the clustering computed by Algorithm 3 is less or equal to the size of the clustering from Algorithm 2.

The function *gnawrooting* processes all edges of the input tree in linear time, thus, the time complexity of the loop from line 11 is equal to $O(\sum _{i}|U_{i}|)$. A similar property has the decomposition from lines 7-9. The ME score for rooted trees is computed by Algorithm 1 two times: in line 10 and in line 12. Hence, the time and space complexity of Algorithm 2 is $O\left (|S|+\sum _{i}|U_{i}|\right)$. □



#### **Lemma 12**

Algorithm *4* computes the upper bound of ME score in $O(|S|+\sum _{i}|U_{i}|)$ time and space.

#### *Proof*

Algorithm 4 returns the number of episodes computed for exactly one set of rootings that uniquely corresponds to an element from the product of classes $U^{\ast }_{1}/_{\sim } \times U^{\ast }_{2}/_{\sim } \times \dots \times U^{\ast }_{n}/_{\sim }$. Hence, this number of episodes is evaluated in $\max $-formula in line 4 of Algorithm 2. Therefore, the ME score computed by Algorithm 2 is bounded above by output of Algorithm 4. The class of the maximal size for a gene tree *G* can be found in *O*(|*G*|) time, therefore, the complexity of the decomposition from lines 3-6 is $O\left (\sum _{i}|U_{i}|\right)$. □

Algorithm 4 is a greedy heuristic in which the method of class selection can be replaced in several ways, e.g., by using a random class, the minimal size class or the class with the minimal value of *gnaw*. Moreover, it could be further refined to obtain a feasible algorithm similar to one presented in [36].

Finally, we present Algorithm 5. It is a heuristic solution to UME Problem having a quadratic time complexity. Algorithm 5 is designed to utilize the following property: if the input consists of thousands of trees, then it is more likely that clustering of duplications from all non-plateau rooted subtrees is sufficient to approximate, or even to provide, the exact ME score. Therefore, Algorithm 5 first solves computationally simple instances of RME extracted from the input gene trees and, then if the solution is not found, it proceeds to complex unrooted parts. In the next Section (see Table 1), we observe a surprising performance of Algorithm 5 allowing to solve exactly hard instances containing a large number of complex classes with runtimes counted in seconds. Also, when the ‘rooted’ part of an instance is small (see the Guigó dataset with 53 trees), the runtime could be much worse than for the large and potentially hard datasets (e.g., Génolevures with 4144 trees).

**Table 1:** Datasets: properties, scores and runtimes

			1 class		3 classes	5 classes	Lower	Upper	ME score	Runtime
Dataset	Size	Species	Double	Empty	empty	empty	bound	bound	(exact)	of
		tree	edge	edge	edge	edge	by Alg.3	by Alg.4	by Alg.5	Alg.5
Guigó	53	*S*_1_ [51]	0	41	12	0	3	7	5	< 30 min
		*S*_2_ [20]	3	38	12	0	3	6	5	< 30 min
TreeFam	1274	NCBI [56]	133	611	463	67	227	227	227	∼ 40 s
Génolevures	4144	[54]	589	2226	1274	55	100	100	100	∼ 40 s
		[55]	673	2250	1079	142	91	91	91	∼ 40 s



#### **Lemma 13**

Algorithm *5* is a heuristic solution to UME that runs in $O\left (\left (|S|+\sum _{i}|U_{i}|\right)^{2}\right)$ time and $O(|S|+\sum _{i}|U_{i}|)$ space.

#### *Proof*

The first part of Algorithm 5 consists of two phases. The first phase (lines 10-11) has a linear time complexity (see Lemmas 11 and 12). In the second phase (lines 12-24) it may provide an exact solution in quadratic time due to the calls of *gnaw*.

In the second part of Algorithm 5, depending on the size of $\mathcal {E}$ it is either computing an exact solution by applying Algorithm 2, or it returns a heuristic solution that has quadratic worst-time complexity. This part of the heuristic is similar to Algorithm 4, however, instead of selecting the largest class we choose the class with the minimal ME score (see line 20).

Observe, that some duplications, which are included in Algorithm 5 in line 12 and corresponding to Algorithm 3 line 9 in Algorithm 5 are included for the second time. Note, the ME score will remain the same, because all of them have a plateau leaf ancestor. □

### Implementation

Our algorithms are implemented in a prototype computer program written in C++ and python. Additionally, for a more detailed output, all score computing algorithms are extended with a routine for the reconstruction of gene duplication clusters (episodes) with their location in the species tree. The software is available on request.

## Results and discussion

In this section we present the result of evaluation of three datasets: Guigó dataset [20], Génolevures [49] and TreeFam [50]. Datasets properties including the size of classes and the runtime are depicted in Table 1.

### Datasets

**Fig. 5:**
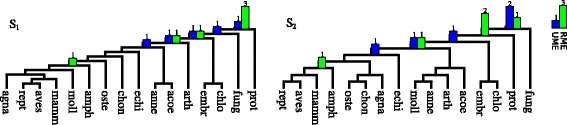
Duplication episodes in Guigó dataset [20] inferred by RME [44] and UME algorithms for the species trees *S*_1_ [51] and *S*_2_ [20]

*Guigó dataset* is a collection of 53 rooted gene trees from 16 Eucaryotes [20]. Multiple gene duplication events were inferred for two species trees: *S*_1_ from [51] and *S*_2_ from [20]. The comparison of the results for RME [44] and Algorithm 2 is shown in Fig. 5, where the original rooting of each gene tree was ignored in UME.

**Fig. 6:**
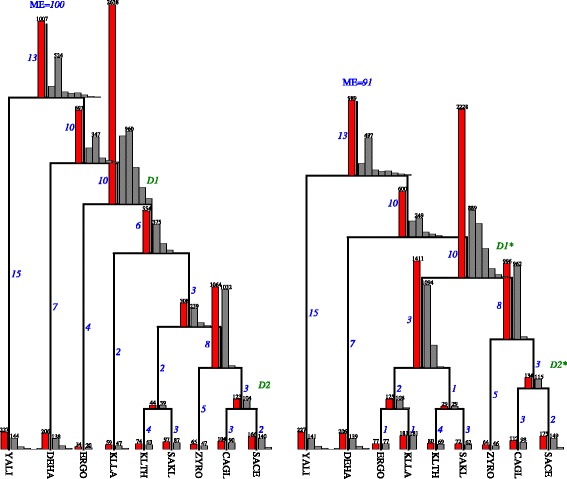
*Left:* a summary of 100 duplication episodes found in Génolevures dataset [49] by Algorithm 5 for the species trees from [54]. *Right:* 91 duplication episodes found in the species tree from [55]. *D*2 and *D*2^∗^ denote one whole genome duplication (WGD) event suggested in [58, 59], while *D*1 and *D*1^∗^ denote one WGD event from [57]. The number of episodes assigned to a single edge is presented on the side (blue italic), for example, our algorithm found 13 duplication episodes in the rooting edge in both trees. A gray histogram (the right side of a node) denotes the frequency of gene trees (*Y* axis) being involved into exactly *x* (*X*-axis starting from 1) episodes located on the corresponding node. The number above the highest bar denotes the maximal number of such gene trees. For example, the gray histogram in the left tree with the second bar of the size 960 denotes that there are 960 gene trees contributing to exactly 2 episodes at the current node. Bars of frequency lower that 10 are not shown. A red bar on the left of a node denotes the number of gene trees having at least one duplication event mapped to this node, i.e., the sum of bars of the corresponding gray histogram

*Génolevures* consists of 4144 gene families from nine yeast genomes [49]. We used the corresponding gene family trees inferred by the authors of [52] using tools from Phylip [53]. The gene trees were reconciled with the species trees from [54] and [55]. The summary of duplication episodes found by our algorithms is depicted in Fig. 6.

**Fig. 7:**
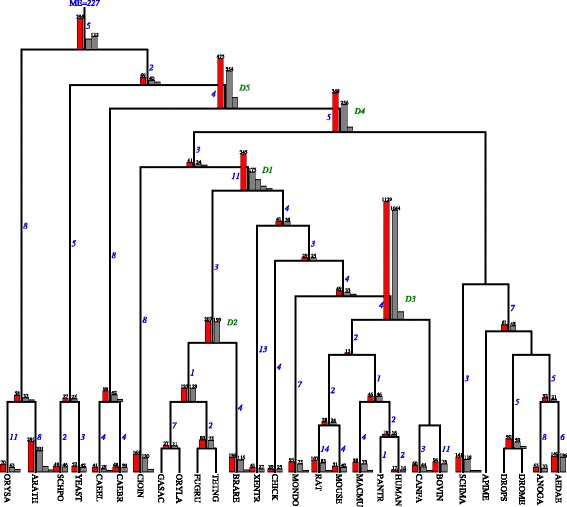
Two hundred twenty seven duplication episodes found by Algorithm 5 for the TreeFam dataset. The upper and lower bounds returned by our algorithms are the same, therefore, 227 is the exact solution. Please refer to Fig. 6 for the description of numbers and histograms. Two consecutive WGD events at *D*1 and one WGD event at *D*2 are reported in [60–62]

*TreeFam* consists of 1274 unrooted gene family trees [50] sampled from 28 mostly animal species. The species tree is based on NCBI taxonomy [56]. The summary of duplication episodes found by our algorithms is depicted in Fig. 7.

### Discussion

*Guigó dataset:* The clustering for the species tree *S*_1_ indicates that UME algorithm found a better scenario than RME, i.e., 5 episodes vs. 6. Additionally, the duplication locations are generally in agreement with the solution to the unrooted variant of episode clustering (see more in [36]). Next, the result of RME for *S*_2_ is consistent with [20, 38]. However, in [37] authors suggested a different evolutionary scenario having more duplication episodes. The results differ, i.e., for the gene tree for *β*-nerve growth factor precursor (NGF) of topology (*rept*,(*mamm*,(*amph*,*aves*))) in the placement of two duplications inferred by that gene tree and *S*_2_. In the optimal solution from UME algorithm, the rooting of NGF gene tree is (*aves*,((*mamm*,*rept*),*amph*)) and it infers one duplication with *S*_2_.

*Génolevures:* We locate two genomic duplication events spanning a large number of gene trees in the left species tree: one situated at *D*1 (2638 trees) and the other above *D*2 (1064 trees). While in the right tree, we have three such events: at *D*1^∗^ (2228 trees) and the children of *D*1^∗^. There is a definite correspondence between the events located above *D*2 and *D*2^∗^. Next, we observe at least 960 trees participating in two duplication clusters at *D*1. Therefore, we postulate that *D*1 has at least two large genomic duplications. Also, they seem to correspond to two events from the right tree located at *D*1^∗^ and the left child of *D*1^∗^.

In comparison to the literature, we claim that the peaks at *D*1 and *D*1^∗^ match the whole genome duplication that was a direct consequence of ancient interspecies hybridization [57]. The location of a WGD event at *D*2 and *D*2^∗^ [58, 59] is not supported by our analysis. Based on UME clustering, the most likely location of such an event is their parent, i.e., the root of (*ZYRO*,(*CAGL*,*SACE*)).

*TreeFam:* The episode clustering (see Fig. 7) indicates several genomic duplications located at *D*1, *D*2, *D*3, *D*4 and *D*5. The dataset have only two plant genomes so it is inadequate to study WGD in plants. The same applies to yeasts (2 species), worms (2 species) and insects (6 species). The major part of TreeFam consists of Chordates, for which various studies [60–62] suggest the existence of two consecutive WGDs located at *D*1 as well as one WGD event at *D*2. Both are partially supported by our analysis by the presence of relatively large number of gene trees contributing to gene duplication events at these two nodes. The genomic duplication at D3 spans almost every tree from the dataset suggesting one WGD event, however, we did not find any evidence of such an event in the literature.

## Conclusions

In this article, we proposed the first solution to the problem of minimum episodes clustering for the case when input gene trees are unrooted. We showed new properties of unrooted reconciliation for the duplication cost. Then, we proposed a decomposition of an unrooted gene tree that allows transforming a gene tree into a set of rooted trees and a simplified unrooted tree. Based on the tree decomposition, we designed several exact and heuristic algorithms for solving the problem. From the application point of view, the most important is an efficient heuristic algorithm, which in practice allows solving exactly empirical instances consisting of thousands of unrooted gene trees. Our evaluation on empirical datasets confirmed several genomic duplication events from the literature.

### Future Work

Future work will focus on the open question of the complexity of UME (we conjecture that UME is intractable). Moreover, we plan to research on the applications of the developed theory to infer genomic duplication events from simulated and empirical datasets of unrooted gene trees including a comparative study of other models of duplication intervals [36].

## Abbreviations

D: Gene duplication
DL: Gene duplication and loss
lca: Least common ancestor
ME: Minimum episodes clustering for rooted gene trees
UME: Minimum episodes clustering for unrooted gene trees
